# The impact of a GP clinical audit on the provision of smoking cessation advice

**DOI:** 10.1186/1447-056X-7-4

**Published:** 2008-10-14

**Authors:** Lisa McKay-Brown, Nicole Bishop, James Balmford, Ron Borland, Catherine Kirby, Leon Piterman

**Affiliations:** 1 Research Fellow, Department of General Practice, Monash University, Building 1, 270 Ferntree Gully Road, Notting Hill, 3168, VIC, Australia; 2 Senior Researcher/Statistician, The Social Research Centre, Level 1, 262 Victoria Street North Melbourne, 3051, VIC, Australia; 3 Senior Research Officer, The Cancer Council Victoria, 1 Rathdowne Street, Carlton, VIC, Australia; 4 Nigel Gray Distinguished Fellow in Cancer Prevention, The Cancer Council Victoria; Professorial Fellow, School of Population Health, University of Melbourne. 1 Rathdowne Street, Carlton, VIC, Australia; 5 Research Fellow, Department of General Practice, Monash University, Building 1, 270 Ferntree Gully Road, Notting Hill, VIC, Australia; 6 Head of School of Primary Health Care, Faculty of Medicine, Nursing and Health Sciences, Monash University, Building 1, 270 Ferntree Gully Road, Notting Hill, VIC, Australia

## Abstract

**Aim:**

To investigate whether participation in a clinical audit and education session would improve GP management of patients who smoke.

**Methods:**

GPs who participated in an associated smoking cessation research program were invited to complete a three-stage clinical audit. This process included a retrospective self-audit of smoking cessation management practices over the 6 months prior to commencing the study, attending a 2.5 hour education session about GP management of smoking cessation, and completion of a second retrospective self-audit 6 months later. Twenty-eight GPs completed the full audit and education process, providing information about their smoking cessation management with 1114 patients. The main outcome measure was changes in GP management of smoking cessation with patients across the audit period, as measured by the clinical audit tool.

**Results:**

The majority of GPs (57%) indicated that as a result of the audit process they had altered their approach to the management of patients who smoke. Quantitative analyses confirmed significant increases in various forms of evidence-based smoking cessation management practices to assist patients to quit, or maintain quitting across the audit period. However comparative analyses of patient data challenged these findings, suggesting that the clinical audit process had less impact on GP practice than suggested in GP's self-reported audit data.

**Conclusion:**

This study provides some support for the combined use of self-auditing, feedback and education to improve GP management of smoking cessation. However further research is warranted to examine GP- and patient-based reports of outcomes from clinical audit and other educational interventions.

## Background

Cigarette smoking is the largest single cause of preventable death and ill health in Australia [1]. It imposes substantial costs on the Australian health care system and the wider community, with smoking-associated morbidity and mortality costing over $21 billion per year [2]. For much of the last 30 years there has been a downward trend in cigarette smoking, however in 2004 approximately 17% of people aged 14 years and over were still smoking on a daily basis [3]. More needs to be done to promote smoking cessation, particularly in general practice, given that approximately 85% of the population visits a general practitioner (GP) at least once per year [4].

Training GPs to effectively deliver smoking cessation advice remains a challenge to health educators. Advice from GPs has a small but statistically significant effect on smoking cessation rates [5]. Yet despite this, the implementation of effective smoking cessation programs in general practice has been difficult to achieve [6]. Barriers cited by GPs include lack of expertise to counsel smokers, limited time to do so and no reimbursement policies for smoking cessation counseling [7]. In addition, GPs appear reluctant to participate in research designed to remedy these deficits [8].

Clinical audits have the potential to be useful for self-assessment and quality improvement in medicine [9] where one aspect of medical care or its provision is assessed over time [10]. While the effectiveness of audit and feedback as a strategy for behaviour change can be variable, [11] a recent meta-analysis found it can be effective in improving professional practice [12]. While in general the effects were small to moderate, efficacy of audit and feedback is likely to be larger when feedback is provided more intensively to GPs and when they are actively involved in implementing change [12]. The timing of feedback delivery and the credibility of the feedback source are also important factors [11].

Auditing the management of smoking can improve both GP management and increase smokers quit rates [13]. This paper describes GP behaviour change that occurred over the duration of a smoking cessation research project that included a clinical audit, combined with education.

The focus of this paper is on the use of established clinical audit processes to review GP management of smoking cessation against explicit criteria set out in the *Smoking Cessation Guidelines for Australian General Practice*, [14] while participating in a larger research study. The aim of the larger study was to compare two approaches to encouraging smoking cessation within general practice: in-practice management versus referral to a specialist evidence-based smoking cessation service.

It was hypothesized that participation in the audit process would produce evidence-based improvements in GP management of patients who smoke, as measured by the clinical audit.

## Methods

### Participants

GPs were recruited to the broader research study via Divisions of General Practice and mail-outs to other GP networks (for full details of recruitment see McKay-Brown et al, 2007). Forty-five GPs participated in the research study and attended the education session. Of these GPs, 28 completed the full audit process. Clinical audit data were obtained for 1114 patients; Audit Part 1 (AP1) n = 558, Audit Part 2 (AP2) n = 556.

### Materials and procedures

The clinical audit tool was developed from recommendations outlined in the *Smoking Cessation Guidelines for Australian General Practice *(the Guidelines). Prior to distribution, audit materials were piloted and reviewed by three GPs to ensure the instructions and data entry sheets were clear and relevant.

GPs received instructions to:

Identify 20 patients aged 18 years and over via Medical Director (or similar), who:

• have attended your practice within the past 6 months (prior to GP attendance at education session); and

• currently smoke, are attempting to quit, or have quit in the last 6 months.

• Examine the past 6 months of clinical notes for each patient, and record the requested information on the Patient Audit Sheets.

GPs were asked to systematically select patients who met the inclusion criteria from their medical records, neither intentionally selecting nor avoiding patients at AP2 who were included in the broader research study. As patient data reported in the clinical audit were anonymous, the number of patients who were included both in the audit and the broader research project are unavailable.

### Audit Part 1

**Figure 1:**
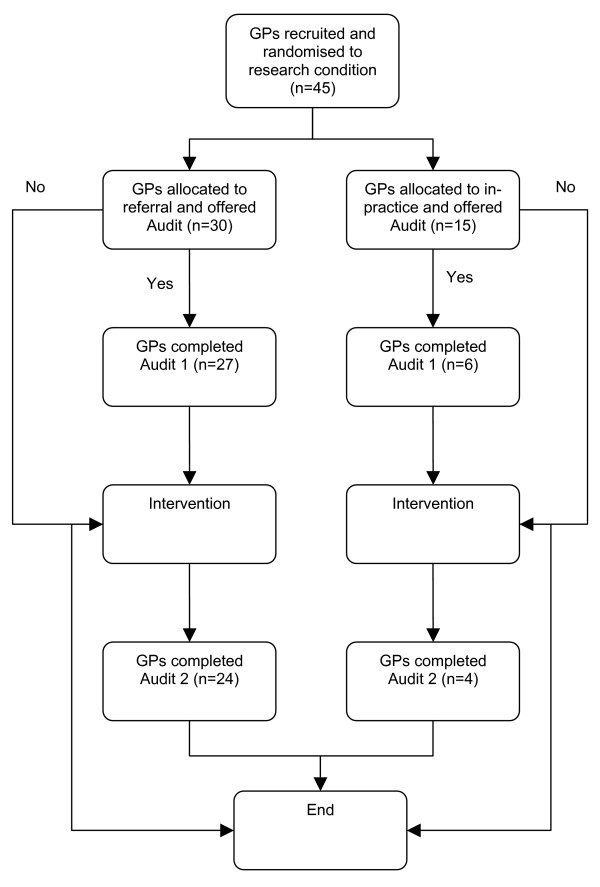
The course of the study – audit process.

GPs completed Audit Part 1 prior to commencing patient recruitment for the intervention study (see figure 1). GPs first completed a six-item *Pre-Audit Questionnaire *about motivation to participate in the audit, factors affecting the provision of smoking cessation advice, and awareness of, and confidence in using the Guidelines to assist patients to stop smoking. GPs then identified 20 patient records that met the eligibility criteria and completed a *Patient Audit Sheet *for each record. The audit sheets required information about patient's smoking history, whether smoking cessation had been raised or discussed, the patient's readiness to change; actions taken by the GP to assist the patient to quit and follow-up on the patient's progress (see Figure 1).

After all audit data was collated, GPs received a one page summary of their own and their colleagues' audit results along with a reflection survey and information on the Guidelines. The survey comprised six questions that required GPs to evaluate their Part 1 results, compare these to the Guidelines, and identify ways in which they could improve their practice.

### Education

GPs attended a 2.5-hour education session presented by trained cessation counsellors. This session provided GPs with information about the Guidelines, including the use of the 5 As (*Ask, Assess, Advise, Assist *and *Arrange *follow-up) for structuring smoking cessation in health care settings. GPs also received specific training in the management option to which they had been randomised (in-practice management or referral) for the associated research study.

### Audit Part 2

Approximately 6 months after completing AP1 and after participation in the research project, GPs received the AP2 pack, which included patient audit sheets and a *Post-Audit Questionnaire*. AP2 was completed using the same criteria for selection as AP1, and involved recording the same information from the patient records. The eight-item *Post-Audit Questionnaire *included questions about the GP's experience of the audit and the perceived impact on their clinical practice.

GPs again received a summary report of their AP2 results with comparative data on colleagues' practices and were asked to complete a final reflection survey.

### Outcomes and Analysis

The main outcome was changes in GP management of patients who smoke, across the audit period, as measured by AP1 and AP2.

As this study used pre-post data, quantitative (weighted) analyses were conducted throughout to account for the paired cluster design, using Stata SE 7 weighted survey techniques. An alpha level of 0.05 was used for all statistical tests. In order to take into account the correlated nature of the data and repeated measures over time, generalized estimating equations (GEE) were used for a final analysis of outcomes. Robust (or empirical) variance was used to compute the p-values for the parameter estimates. Qualitative responses collected from the pre-audit questionnaires and GP reflections were coded and analysed thematically.

## Results

### GP feedback on the audit process

GP's most commonly cited reasons for taking part in the audit were to enhance their knowledge and skills (42%), to help patients quit (30%), to receive Continuing Professional Development (CPD) points (16%) and to address a public health concern (12%). The majority indicated that they wished to increase their skills in the provision of smoking cessation advice.

After audit completion, 68% of GPs reported that the audit process met their learning needs and 71% believed that completing the audit had assisted their management of patients who wished to quit. Over half of GPs (57%) indicated that as a result of the audit process they had altered their approach to the management of patients who smoke.

### Changes in GP practices

The average age of patients included in the audit was 42 (SD = 15.4) years and a greater proportion (56.9%) were female. There were no significant gender or age differences between AP1 and AP2.

As displayed in Table 1, there was a significant increase in evidence-based smoking cessation management practices across the two audits. The issue of smoking was raised with a greater percentage of identified smokers, and more assistance was provided at AP2. This included significant increases in GP provision of assistance during consultations; provision of advice on, or prescription of medication; and referral to specialist cessation services. There was also an increase in the use of follow-up in subsequent consultations from 24% to 35% (see Table 1).

**Table 1:** Types of cessation actions employed by GPs, by audit (n = 1,114)

*Characteristics*	*Audit 1 (n = 558)*	*Audit 2 (n = 556)*	*p*
Raised issue of smoking cessation	74%	87%	0.035
Taken action to assist patient to quit	79%	89%	0.005
Provided patients with clear advice to quit	53%	70%	0.001
Provided additional help within consultation†	14%	23%	0.006
Provided printed material	22%	38%	0.003
Provided product advice (NRT or Bupropion)	12%	19%	0.006
Referred patients to other services	10%	22%	0.002
Followed up and reviewed progress	11%	21%	0.019

† discussed barriers to quitting, CBT strategies or assisted patient to devise a quit plan

Actions employed by GPs to assist patients to quit were also analysed using only the records of patients where the issue of smoking had been raised during the consultation (n = 893; 80%). As displayed in Table 2, the frequency of GP-initiated discussion about smoking cessation did not increase across the audit period. However, when the issue was raised, the audit data indicated that GPs began to take more detailed patient histories (see Table 2).

**Table 2:** Proportion of patients where cessation was raised in consultation, by audit (n = 893)

*Characteristics*	*Audit 1*	*Audit 2*	*p*
GP initiated cessation discussion	80%	78%	0.566
Assessed readiness to quit	94%	97%	0.008
Asked about previous unsuccessful attempts	40%	54%	0.002
Assessed nicotine dependence‡	38%	68%	0.001

‡ number of cigarettes per day and minutes after waking until first cigarette

### Patient reports of GP management

As a partial test of whether the audit process or the education session was primarily responsible for the changes in GP practice, ancillary analysis were performed using patient data from the associated research study. Patient data regarding GP practices were compared between GPs who completed the audit and those who did not (Table 3). The data used for these analyses comes from the intervention study in which there was 2:1 random allocation to referral versus in-practice conditions [8]. After controlling for the intervention to which GPs were randomised, based on patient data, we found no significant differences between GPs who did and did not complete the audit on raising the issue of smoking cessation, providing smoking cessation advice to patients, or discussing pharmacotherapies. However, GPs who completed the audits were significantly more likely to discuss the use of Quitline with their patients, when compared with the non-audit GPs (see Table 3).

**Table 3:** Smoking cessation advice provided, by audit and non-audit GPs

*Characteristics*	*Non-audit GPs *(n = 17)	*Audit GPs *(n = 28)	p*
GP discussed smoking	75%	83%	0.335
GP provided advice to cut down/quit	43%	46%	0.815
GP spoke about medication	36%	38%	0.820
GP discussed use of Quitline	51%	70%	0.001

controlling for the intervention

## Discussion

Completing the audit cycle allowed GPs to observe their recorded behaviour at AP1, compare it with expected standards and the behaviour of peers (standardised feedback), and evaluate change in their own behaviour following participation in the education session and research study.

Results from the clinical audit provides some initial and encouraging support for the combined use of audit, individualised feedback and face-to-face education to improve GP provision of health care advice. GPs reported improvements in discussion of smoking cessation within a consultation, collecting more information on smoking history and current readiness to change, and providing more cessation advice, and/or more frequent referral to a specialist cessation service.

In addition, findings from GPs' post-audit questionnaires and reflection surveys indicated increased use and understanding of smoking cessation guidelines, and enhanced knowledge and skills pertaining to smoking cessation management. These results were encouraging and suggested that audit and feedback, within a broader intervention study, could lead to improvements in the provision of health care advice.

However, further analyses of patient data from the broader intervention study challenge these findings, providing contrasting results to the GP self-reported data. Patient data indicated that aside from more frequent discussions of Quitline with patients, there were no other significant differences between GPs who did or did not complete the clinical audit. Trends favoured GPs who completed the audit, suggesting that audit participation had some impact on other evidence-based smoking cessation practices undertaken by GPs. Despite this, the patient data clearly suggest that the measured positive changes in GP learning and behaviour may be in fact more attributable to participation in the education session and intervention study than the audit process alone. This supports previous research findings that interventions that have multiple components and are practice-based are more effective in changing practitioner behaviour and improving patient quit rates [13].

This post-hoc analysis of patient data from the corresponding intervention study yielded unexpected results, yet provide some confirmation of the common criticism that self-reports of activity tend to overestimate actual performance, [16] and this may have occurred with the audit process. Further research is clearly warranted to further compare GP and patient-based data during a process of clinical audit interventions, both individually and in combination with other educational interventions. We do not believe that the current data discredit the use of clinical audit, but that further examination is required to disentangle the biases of self-report from the benefits that the self-auditing process can have on clinical practice.

Some limitations must be noted. Firstly, through considered, systematic selection of patients, AP2 may have contained cases that were part of the broader intervention study and this might have also contributed to improved GP management of those cases. Secondly, we do not know whether the improvements in management were sustained beyond the initial period after the education session, regardless of how the improvements occurred. Finally, only a small proportion of GPs were prepared to be part of the research study, and fewer still were prepared to participate in the audit. It may be that this motivated group have used the opportunity to improve their practice, but it is uncertain whether less motivated GPs would similarly improve even if they could be encouraged to participate in such a process.

## Conclusion

This study provides some support for the combined use of clinical audit and education to improve GP management of smoking cessation, however patient data challenged the GP self-reports of practice improvement across the audit period. Further research is clearly warranted to examine the effectiveness of clinical audit using both GP- and comparative patient-based data. Findings from this study show that involvement in multi-component research can support GPs to consider the issues of smoking cessation more fully, particularly the taking of detailed patient histories and providing advice and referral. In areas of general practice where there is an identified need to improve practice, encouraging greater use of clinical audit in association with other forms of education may be an effective strategy for improving standards of patient management. As getting their smoking patients to stop is probably the single most important thing a GP can do to improve those patients future health prospects, encouraging more GPs to audit their performance in this area, and undertaking education sessions in how to better manage their patients would seem to be a high priority.

### Summary of implications for GPs

Smoking cessation management continues to be an important part of the health prevention and intervention activities of GPs. With research showing that advice from GPs has a small but statistically significant effect on smoking cessation rates, finding ways to effectively train GPs to deliver smoking cessation advice remains a challenge. This manuscript provides further evidence that combined audit and feedback with education can encourage behavioural change.

## Authors' contributions

LMB conducted the audit component of the study, managed the data, and drafted and revised the manuscript, NB performed the statistical analysis and helped to draft and revise the manuscript, JB participated in the design of the study, conducted the intervention component and helped to draft and revise the manuscript, RB and LP conceived of the overall study, and participated in its design and coordination and helped to draft and revise the manuscript, CK developed the audit materials used in this study and helped to draft and revise the manuscript. All authors read and approved the final manuscript.
